# Analysis of risk factors for anastomotic leakage after radical esophagectomy for esophageal squamous cell carcinoma

**DOI:** 10.3389/fmed.2025.1668790

**Published:** 2025-10-31

**Authors:** Qing Hu, Lu Sun, Shijie Xu, Weidong Hong, Liangcheng Tang, Feng Li, Yougui Wang

**Affiliations:** ^1^Department of Thoracic Surgery, Affiliated Chuzhou Hospital of Anhui Medical University (The First People’s Hospital of Chuzhou), Chuzhou, Anhui, China; ^2^Department of Oncology, Affiliated Chuzhou Hospital of Anhui Medical University (The First People’s Hospital of Chuzhou), Chuzhou, Anhui, China; ^3^Department of Pathology, Affiliated Chuzhou Hospital of Anhui Medical University (The First People’s Hospital of Chuzhou), Chuzhou, Anhui, China; ^4^Department of Thoracic Surgery, Affiliated Hospital of Jiangsu University, Zhenjiang, Jiangsu, China

**Keywords:** anastomotic leakage, esophageal squamous cell carcinoma, esophagectomy, anastomotic location, tension, risk factor

## Abstract

**Objective:**

Anastomotic leakage (AL) is one of the most common complications of radical surgery for esophageal cancer. This study aimed to analyze the risk factors for AL after radical esophagectomy for esophageal squamous cell carcinoma (ESCC) and construct a nomogram prediction model.

**Methods:**

We retrospectively analyzed the clinical data of all patients who underwent radical esophagectomy between 2018 and 2023. Univariate and multivariable logistic regression analyses were used to identify the risk factors for AL. After screening the relevant variables, a prediction model for AL risk was established, and the predictive accuracy and clinical utility of the model were verified.

**Results:**

A total of 107 patients with ESCC were included and the incidence of AL was 21.5% (23/107). In multivariate logistic regression analysis, age (OR 1.131, 95% CI 1.014–1.261, *p* = 0.027), anastomotic location (OR 5.747, 95% CI 1.754–18.828, *p* = 0.004), postoperative red blood cell (RBC) (OR 0.152, 95% CI 0.042–0.543, *p* = 0.004), and postoperative neutrophil to lymphocyte ratio (NLR) level (OR 1.096, 95% CI 1.017–1.182, *p* = 0.016) were considered as independent risk factors for the occurrence of AL. Based on the results of the multivariate logistic regression analysis, a nomogram was constructed, and the area under the receiver operating characteristic (ROC) curve (AUC) was0.870. The decision curve analysis (DCA) demonstrated the clinical utility of this model.

**Conclusion:**

Age, anastomotic location, postoperative RBC count, and postoperative NLR were independent risk factors for AL after radical esophagectomy for ESCC. In addition, this study innovatively provides the mechanistic hypothesis linking cervical AL to the combined effects of anastomotic tension and impaired perfusion, offering a pathophysiological basis for its higher incidence than thoracic anastomosis.

## 1 Introduction

Esophageal cancer is a malignant tumor that seriously threatens human health and is characterized by high morbidity and mortality (1). Radical esophagectomy is the preferred treatment for resectable esophageal cancer (2). In recent years, with the continuous development of surgical procedures, and improvements in perioperative management, the incidence of postoperative complications of esophageal carcinoma has decreased, however, AL is still one of the most serious postoperative complications. The incidence of anastomotic leakage has been reported to vary in different studies, ranging from about 4.9 to 41% (3, 4).

AL can lead to decrease quality of life and increase risk of death in patients (5). If AL cannot be detected early and treated aggressively, patients may experience further complications such as pleural effusion, pneumothorax, mediastinal infection, empyema, and even death (6). Common risk factors for AL include gender, age, hypoproteinemia, diabetes, and overweight. Additionally, factors such as anastomotic modality, anastomotic position, and tension are closely related to the occurrence of AL (3, 6, 7). Therefore, it is crucial to identify early high-risk populations for AL, and use accurate predictive tools for early diagnosis and prevention. This study aimed to explore the risk factors for AL after radical esophagectomy in patients with ESCC and to establish a nomogram model for predicting anastomotic leakage, which will provide clinical evidence for early clinical prevention and treatment of AL.

## 2 Methods

### 2.1 Patient selection and data collection

This study enrolled patients who underwent radical surgery for esophageal cancer on the southern campus of the Affiliated Chuzhou Hospital of Anhui Medical University between 2018 and 2023. All patients underwent preoperative electronic gastroscopy, and the diagnosis of an esophageal malignant tumor was confirmed. They did not undergo any therapeutic endoscopic procedures. This retrospective study was approved by the Medical Ethics Committee of the affiliated Chuzhou Hospital of Anhui Medical University (Approval No. 2024–026) and was conducted in accordance with the 1964 Helsinki Declaration and its later amendments or comparable ethical standards. Informed consent was waived by our Institutional Review Board because of the retrospective nature of our study.

The inclusion criteria were as follows: 1. preoperative clinical diagnosis of esophageal cancer and completion of esophageal cancer surgery between 2018 and 2023; 2. complete clinical data without missing information; 3. Absence of distant metastases or other histories of malignancy. The exclusion criteria were as follows: 1. patients who did not undergo radical esophagectomy, or underwent salvage or palliative surgical procedures; 2. intraoperative death; 3. pathology indicating non-squamous cell carcinoma; 4. incomplete clinical data. Patients were divided into two groups based on the occurrence of AL postoperatively: AL and NAL groups.

Based on previous studies and clinical experience, we collected and analyzed the following patient-related data: gender, age, weight, American Society of Anesthesiologists (ASA) classification, history of underlying diseases (hypertension, diabetes), smoking history, alcohol history, anastomotic location, extent of tumor invasion, pathological T and N category (according to the eighth edition AJCC TNM staging system), tumor location, whether preoperative neoadjuvant therapy was received, preoperative and postoperative day-three level of white blood cell (WBC), RBC, hemoglobin (Hb), albumin (ALB), NLR, PLR, LMR, C-reactive protein (CRP)/ALB, and OPNI. Laboratory tests were uniformly collected at 6:00 a.m. on postoperative day 3, following overnight fasting. All assays were performed in the hospital’s central laboratory using standardized protocols. The thresholds for abnormal values followed institutional standards.

### 2.2 Surgical procedure

All patients underwent standard surgical treatment for esophageal cancer (McKeown, Ivor-Lewis, or Sweet esophagectomy). Intrathoracic or cervical anastomosis was established using a mechanical procedure. The choice of surgical procedures was primarily determined by tumor location, combined with consideration of the surgeon’s clinical experience. Minimally invasive techniques were planned for nearly all patients, with conversion to open procedure occurring only if intraoperative findings compromised procedural safety. In our standard practice, all patients undergoing esophagectomy receive intraoperative feeding tube placement. All procedures were performed by the consistent surgical team, and perioperative management was highly standardized. In addition, enhanced recovery protocols were not systematically implemented during the study period.

### 2.3 Diagnosis of AL

AL is defined as a full thickness GI defect involving the esophagus, anastomosis, staple line, or conduit, irrespective of the presentation or method of identification (8). The primary diagnostic methods for AL include contrast swallow (esophagography), CT scanning, and upper gastrointestinal endoscopic examination, which often reveal mediastinal effusion and pneumomediastinum, wall discontinuity at the anastomotic site, or fistula. In addition, based on our experience and previous studies (6, 9), we considered the occurrence of a hidden thoracic fistula in the presence of specific clinical manifestations and laboratory tests, such as persistent hyperthermia and significantly increased inflammatory markers.

In this study, all patients underwent imaging examinations on postoperative day 7 or at symptom onset to ascertain the presence or absence of AL.

### 2.4 Definitional criteria and calculation formulas

NLR = The neutrophil to lymphocyte ratio.

LMR = The lymphocyte-to-monocyte ratio.

PLR = The platelet to lymphocyte ratio.

The OPNI (Onodera Prognostic Nutrition Index) value = ALB value (g/L) + 5 × total lymphocyte number (10^9^/L).

### 2.5 Statistical analysis

Data differences were analyzed using the SPSS software (version 26.0). Quantitative data between the AL and NAL groups were statistically analyzed using the *t*-test or Mann–Whitney U-test. Qualitative data were compared using *χ*^2^ test. Variables with *p* < 0.05 in univariate analysis were entered into the multivariable logistic regression analyses, and independent risk factors for AL were identified (*p* value< 0.05 was considered statistically significant). A predictive model for the risk of AL was developed using the “nomogram” function based on the “rms” package of the R software (version 3.6.0). The area under the ROC curve (AUC) was used to verify the effectiveness of the predictive model in this study. Additionally, the “rmda” package of the R software was employed to draw the DCA to further assess the clinical practicality of the nomogram.

## 3 Results

### 3.1 Patient characteristics

**Figure 1:**
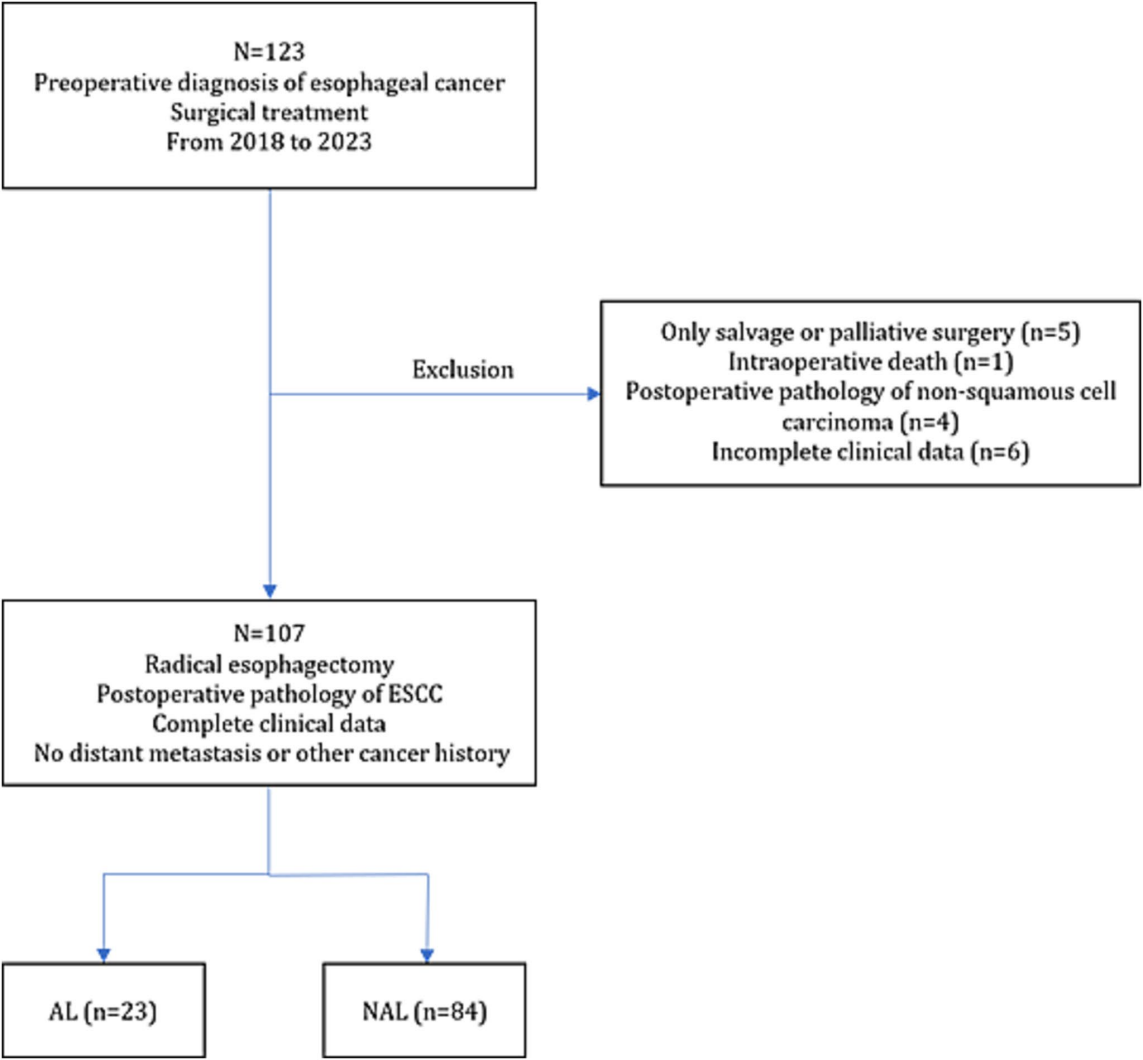
Flowchart of inclusion and exclusion criteria.

A total of 107 patients were enrolled in this study, and the specific inclusion and exclusion flowchart is shown in Figure 1. All 107 patients had ESCC histologically. AL occurred in 23 patients with an incidence rate of 21.5%. The distribution of surgical procedures was as follows: Sweet procedure (3 cases), Ivor-Lewis esophagectomy (67 cases), and McKeown procedure (37 cases). Patient general characteristics, tumor-related data, perioperative infections and nutrition metrics are presented in Table 1.

**Table 1:** Baseline characteristics of patients between two groups.

Characteristics	AL (*n* = 23)	NAL (*n* = 84)	*p* value
Gender			
Female	4	22	0.55
Male	19	62	
Age (years)	69.00 ± 5.75	65.56 ± 7.09	0.035
Hypertension			
Yes	11	26	0.132
No	12	58	
Diabetes			
Yes	4	6	0.275
No	19	78	
Smoking history			
Yes	12	37	0.488
No	11	47	
Alcohol history			
Yes	12	35	0.368
No	11	49	
Weight (kg)	62.54 ± 9.76	62.93 ± 9.24	0.861
Neoadjuvant therapy			
Yes	1	6	
No	22	78	0.996
Anastomotic location			
Cervical anastomosis	14	23	
Thoracic anastomosis	9	61	0.003
ASA			
I–II	20	75	>0.999
III	3	9	
Tumor length (cm)	3.71 ± 2.00	3.46 ± 1.41	0.502
pT category			
1–2	9	35	0.827
3–4	14	49	
pN category			
0–1	21	70	0.535
2–3	2	14	
Tumor location			
Upper esophagus	3	3	0.208
Middle esophagus	9	39	
Distal esophagus	11	42	
Preoperative WBC (×10^9^/L)	6.13 ± 1.65	5.76 ± 1.45	0.296
Preoperative RBC (×10^12^/L)	4.07 ± 0.59	4.38 ± 0.53	0.019
Preoperative Hb (g/L)	125.04 ± 18.32	133.30 ± 15.39	0.031
Preoperative ALB (g/L)	40.57 ± 3.42	42.29 ± 3.58	0.041
Preoperative NLR	2.08 ± 0.72	2.37 ± 0.96	0.179
Preoperative PLR	125.61 ± 35.97	135.79 ± 51.80	0.378
Preoperative LMR	2.97 ± 1.35	3.67 ± 1.41	0.036
Preoperative CRP/ALB	0.05 ± 0.08	0.06 ± 0.14	0.892
Preoperative OPNI	49.33 ± 3.80	50.21 ± 4.53	0.396
Postoperative WBC (×10^9^/L)	14.42 ± 3.18	11.43 ± 3.47	<0.001
Postoperative RBC (×10^12^/L)	3.40 ± 0.59	3.83 ± 0.45	<0.001
Postoperative Hb (g/L)	112.61 ± 11.93	118.40 ± 16.38	0.116
Postoperative ALB (g/L)	35.17 ± 4.18	36.15 ± 4.27	0.328
Postoperative NLR	15.76 (10.29, 20.12)	10.13 (7.48, 13.89)	<0.001
Postoperative PLR	226.32 (132.86, 286.30)	208.46 (161.56, 294.54)	0.994
Postoperative LMR	1.00 ± 0.58	1.63 ± 1.29	0.025
Postoperative CRP/ALB	3.91 (2.65, 4.58)	2.65 (1.57, 3.53)	0.039
Postoperative OPNI	39.35 ± 4.52	41.23 ± 5.58	0.14

Values are presented as mean ± standard deviation or percentiles.

### 3.2 Univariate and multivariate logistic regression analysis

Univariate logistic regression analysis (Table 2) showed 11 potential risk factors associated with the occurrence of AL: age, anastomotic location, and some preoperative laboratory results, including RBC, Hb, ALB, LMR and some postoperative laboratory results, including WBC count, RBC count, NLR, LMR, and CRP/ALB. These risk factors were incorporated into a multivariate logistic regression model (Table 2), age (OR 1.131, 95% CI 1.014–1.261, *p* = 0.027), anastomotic location (OR 5.747, 95% CI 1.754–18.828, *p* = 0.004), postoperative RBC count (OR 0.152, 95% CI 0.042–0.543, *p* = 0.004), and postoperative NLR (OR 1.096, 95% CI 1.017–1.182, *p* = 0.016) were determined to be independent risk factors for the occurrence of AL.

**Table 2:** Univariate and multivariate analysis for risk factors of AL.

Characteristics	Univariate			Multivariate		
	OR	95% CI	*p* value	OR	95% CI	*p* value
Gender	1.685	0.516–5.501	0.387			
Age (years)	1.094	1.005–1.191	0.038	1.131	1.014–1.261	0.027
Hypertension	2.045	0.799–5.234	0.136			
Diabetes	2.737	0.702–10.673	0.147			
Smoking history	1.386	0.550–3.493	0.489			
Alcohol history	1.527	0.605–3.855	0.370			
Weight (kg)	0.996	0.947–1.047	0.860			
Neoadjuvant therapy	0.591	0.068–5.171	0.635			
Anastomotic location	4.126	1.572–10.829	0.004	5.747	1.754–18.828	0.004
ASA	1.250	0.309–5.052	0.754			
Tumor length (cm)	1.106	0.826–1.438	0.498			
pT category	1.111	0.433–2.853	0.827			
pN category	0.476	0.100–2.266	0.351			
Tumor location	0.728	0.342–1.550	0.410			
Preoperative WBC (×10^9^/L)	1.167	0.873–1.561	0.298			
Preoperative RBC (×10^12^/L)	0.336	0.132–0.854	0.022			
Preoperative Hb (g/L)	0.969	0.941–0.998	0.036			
Preoperative ALB (g/L)	0.868	0.755–0.997	0.045			
Preoperative NLR	0.676	0.382–1.097	0.179			
Preoperative PLR	0.995	0.985–1.006	0.376			
Preoperative LMR	0.647	0.425–0.985	0.042			
Preoperative CRP/ALB	0.766	0.017–34.537	0.891			
Preoperative OPNI	0.954	0.857–1.063	0.393			
Postoperative WBC (×10^9^/L)	1.274	1.103–1.470	0.001			
Postoperative RBC (×10^12^/L)	0.174	0.061–0.494	0.001	0.152	0.042–0.543	0.004
Postoperative Hb (g/L)	0.974	0.942–1.007	0.119			
Postoperative ALB (g/L)	0.946	0.847–1.057	0.326			
Postoperative NLR	1.105	1.039–1.175	0.001	1.096	1.017–1.182	0.016
Postoperative PLR	1.002	0.999–1.005	0.170			
Postoperative LMR	0.230	0.083–0.633	0.004			
Postoperative CRP/ALB	1.327	1.023–1.721	0.033			
Postoperative OPNI	0.927	0.839–1.024	0.135			

### 3.3 Construction and validation of the nomogram

**Figure 2:**
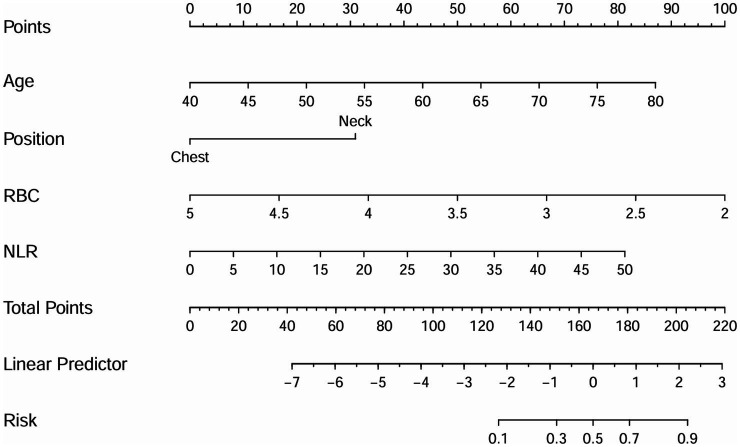
A nomogram for predicting the risk factors of AL in the ESCC patients. To use the nomogram, locate the patient’s value on each variable axis and draw a vertical line upward to determine the points for each variable. The sum of these points is then located on the Total Points axis, from which a vertical line is drawn downward to the Risk axis to determine the probability of anastomotic leakage occurring after esophagectomy.

**Figure 3:**
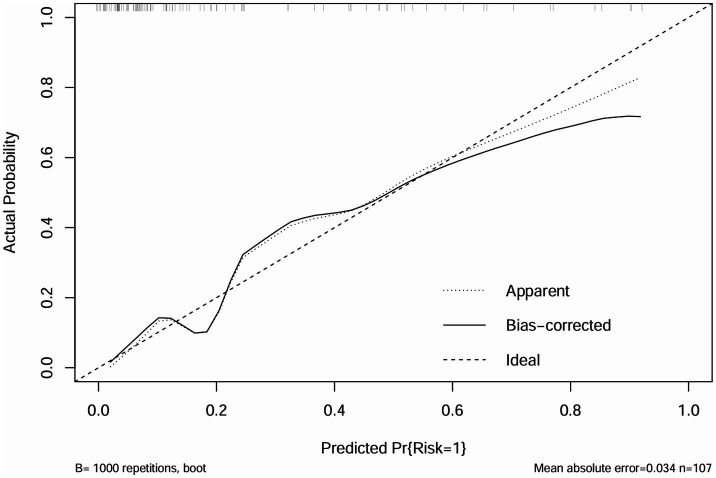
Calibration curve of the nomogram.

**Figure 4:**
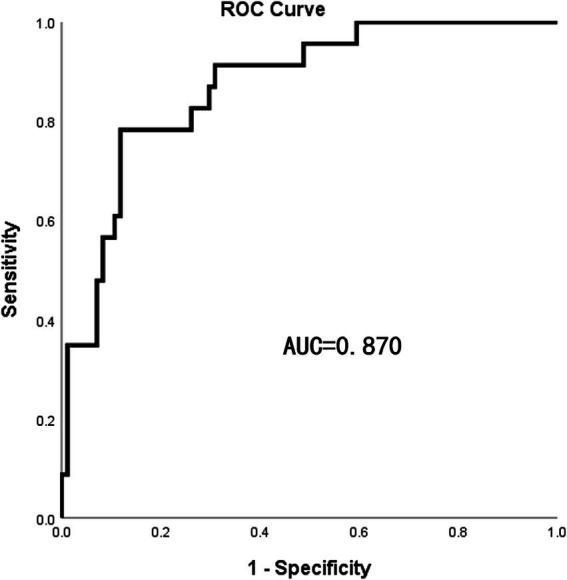
The ROC curve was established. The AUC was used to evaluate the discrimination of the model.

**Figure 5:**
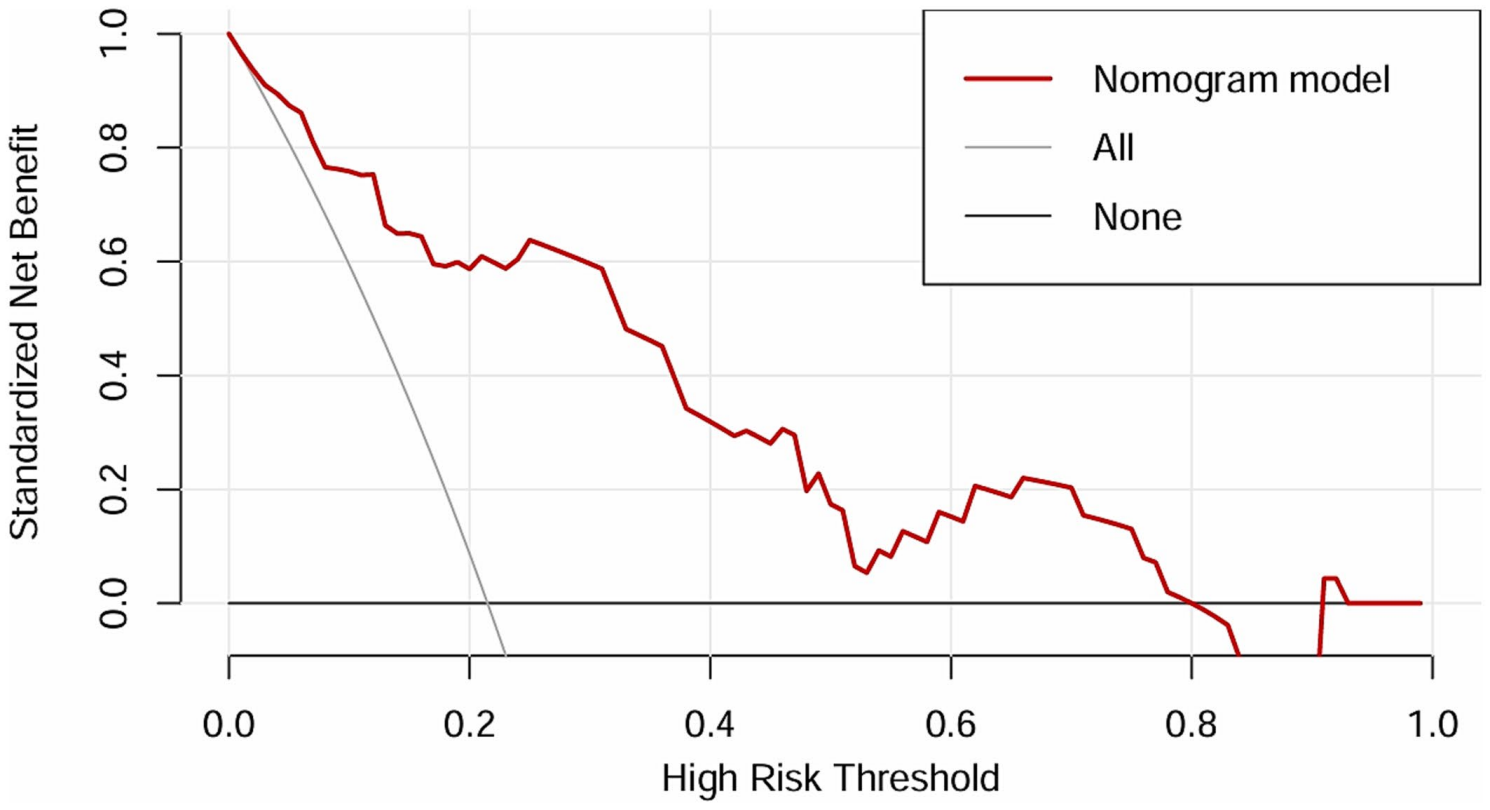
DCA of the nomogram.

The independent risk factors identified from the multivariate logistic analysis were used to construct a predictive model for the risk of AL, which was presented as a nomogram (Figure 2). In this study, the calibration curve of the nomogram for predicting AL risk after esophagectomy for ESCC exhibited good consistency (Figure 3). The accuracy and reliability of the risk model were verified using the AUC of the ROC curve (AUC = 0.870) (Figure 4). DCA was used to assess the clinical utility of the nomogram model (Figure 5).

## 4 Discussion

Esophageal cancer is a common malignant tumor of the gastrointestinal tract, with approximately 90% of ESCC cases in China (10). Surgical resection plays an important role in the treatment of esophageal cancer. AL is one of the most severe complications of esophageal cancer surgery and has a high mortality rate. Despite the increasing number of studies on AL, its pathophysiology and definite causative factors are still unclear. Identifying the risk factors for AL is important for its early prevention and treatment. The main objective of this study was to identify the risk factors for anastomotic leakage and construct a relevant predictive model. Our research indicated that the incidence of AL after radical esophagectomy in patients with ESCC was 21.5%, which is generally consistent with previous reports.

Similar to previous studies, the significance of age in predicting the incidence of AL is contentious (3, 11). In this study, we found that as the age of the patients increased, the incidence of AL also increased. The possible mechanism behind this result is the progressive aging of patients, leading to diminished physiological function, decreased metabolic capacity, higher ECOG score and impaired cardiopulmonary function, resulting in lower surgical tolerance compared to younger individuals. Additionally, the speed of blood supply recovery to the surgical area is relatively slow, requiring more time to recover, which may cause local anastomotic leakage owing to insufficient blood supply (12). Although there is still no consensus on whether older patients are at a greater risk for AL, the majority of researchers believe that advanced age increases the risk of AL after surgical resection for esophageal cancer. Therefore, the issue of postoperative AL in older patients with high ECOG score should be considered seriously by clinicians, warranting special attention in clinical diagnosis and treatment.

At present, it has been confirmed that NLR, as a novel inflammatory marker, can serve as a predictive indicator of postoperative complications and prognosis in tumors (13–15). Under normal conditions, the NLR is low (15), but in cancer patients, lymphopenia and neutrophilia are associated with poor survival (16). Currently, the NLR is primarily applied in the diagnosis and assessment of disease severity in infectious diseases, acute coronary syndrome, and malignant tumors. A meta-analysis by Templeton et al. included 100 studies and explored the effect of NLR on tumor prognosis, revealing that a high NLR was correlated with poor overall survival in many solid tumors, including esophageal cancer (17). A study by Radulescu et al. included 204 patients with gastric cancer, showing that a higher preoperative NLR was significantly associated with the incidence of AL and patient mortality (15). Cook et al. demonstrated that for patients undergoing colectomy, a postoperative NLR level≥ 9.3 on the first day is associated with an increased risk of complications (18). In this study, the postoperative NLR in the AL group was significantly higher than that in the NAL group, and a high postoperative NLR was significantly correlated with the occurrence of AL after surgical resection for ESCC, proving to be an independent risk factor for predicting this complication, which is consistent with the results from several other studies. The optimal cutoff values for the postoperative NLR in this study were confirmed using a receiver operating characteristic (ROC) curve. Controlling postoperative NLR within 14.62 could protect patients from AL and reduced the probability of AL significantly.

Postoperative NLR was significantly elevated in patients with AL. A possible mechanism is as follows. After surgery, large numbers of lymphocytes, neutrophils, and macrophages were gathered at the anastomotic site. Neutrophils are the first inflammatory cells to be attracted and activated at the anastomotic site, releasing proteases and reactive oxygen species during anastomotic healing (19). Low concentrations of reactive oxygen species can promote wound healing, however, the sustained accumulation of high concentrations of reactive oxygen species can increase oxidative stress and lipid peroxidation, leading to severe cellular damage which is detrimental to wound healing (20). Additionally, lymphocytes can regulate the healing process by synthesizing extracellular matrix and remodeling collagen. Inhibition of lymphocyte expression in peripheral blood can lead to impaired collagen synthesis in the extracellular matrix and a decreased capacity for tissue healing (15). Increased NLR after esophagectomy for ESCC may predict a higher incidence of AL.

In this study, low postoperative RBC count was closely associated with the occurrence of AL. There is currently no consensus on the mechanism between anemia and AL. On the one hand, anemia is associated with anastomotic leakage and has been reported in some reports (21–23). Anemia is a pathological condition characterized by a decreased number of circulating red blood cells and a reduced hemoglobin concentration. In this study, low postoperative hemoglobin concentration was statistically significant only in univariate analysis, while it was not included in the model construction of multivariate logistic regression analysis, possibly because of the influence of confounding factors. The primary physiological functions of RBC include oxygen transport, maintenance of systemic acid–base balance, and tissue protection (24). Each stage of wound healing requires an increased oxygen uptake. Research has shown that hypoxia initially acts as a physiological signal to promote wound healing, but prolonged hypoxia can inhibit wound healing, mainly because oxygen plays a crucial role in promoting wound healing by facilitating angiogenesis, collagen synthesis, and epithelialization (25). A reduction in postoperative RBC count means decreased oxygen-carrying capacity of the blood, affecting tissue perfusion and oxygenation, leading to ischemia and necrosis around the anastomosis, which can consequently result in AL. On the other hand, some scholars also think that anemia is not related to the occurrence of AL. Through prospective trials (26), they have found that no significant association was observed between intraoperative tissue oxygenation at the anastomotic site and subsequent AL among patients undergoing Ivor Lewis esophagectomy. They also have proposed that future studies measuring serial postoperatively tissue oxygenation may reveal compromised tissue oxygenation resulting from arterial perfusion deficiency and/or venous congestion, which could contribute to AL. Therefore, actively improving tissue perfusion and preventing anemia postoperatively may help further reduce the occurrence of AL, which requires more prospective studies to clarify.

There is controversy over whether cervical anastomosis is more likely to result in AL than thoracic anastomosis. Some scholars think that thoracic anastomosis might be a better anastomotic approach than cervical anastomosis, with a lower incidence of AL (27). However, a meta-analysis involving data from 13 centers showed that AL was more likely to occur in the cervical group than in the thoracic group (pooled odds ratio = 4.73, 95% CI, 1.61–13.9, *p* = 0.005) (28). Similar results were obtained in another meta-analysis (29). It is generally accepted that higher tension of the cervical anastomosis, insufficient blood perfusion at the anastomotic site, and the requirement for a longer gastric tube in the cervical anastomosis can contribute to a higher risk of AL. Currently, no convincing animal experiments or clinical studies have confirmed that tension affects anastomotic healing. Cui et al. (30) constructed a rat model to study the effect of tension on anastomotic healing and found that the incidence of AL increased with increasing tension, as measured using a tensiometer. Another study on intestinal anastomosis reported that anastomotic tension was an independent predictor of leakage (31), but Katory et al. (32) refuted the relationship between tension and anastomosis, suggesting that increased tension does not cause a higher incidence of leakage. Furthermore, the assessment of tension is often derived from visual estimations by surgeons, which is quite subjective (33), thus, we need more accurate protocols for assessing tension, which may become a focus of future research.

However, studies indicate that insufficient blood perfusion at the anastomotic site is also an important factor affecting the integrity of postoperative esophagogastric anastomosis. Increasing blood flow to the anastomosis after esophagectomy and reconstruction using a variety of modalities can reduce leakage rates from an initial 25% to less than 6% (34). Some researchers have employed indocyanine green fluorescence imaging to evaluate blood flow in the gastric conduit after esophagectomy, allowing timely intraoperative interventions to improve blood perfusion at the anastomosis, thereby reducing the incidence of leakage (35–37). In the reconstructed gastric tube, the right gastroepiploic artery mainly supplies blood (38), however, cervical anastomosis requires a longer gastric tube for gastrointestinal reconstruction, leading to insufficient blood perfusion and poor blood supply, resulting in leakage. Furthermore, as the gastric tube ascends to the cervical anastomotic site, it must traverse the thoracic inlet, where the constricted area may compress the blood-supplying arteries of the gastric tube, affecting its blood supply to various extents and increasing the risk of AL.

Similar to previous studies, we used a simpler and more intuitive nomogram and DCA curve to predict the incidence of AL Multivariable logistic regression analysis showed that age, postoperative NLR, postoperative RBC count, and anastomotic location were independent risk factors for AL, and the constructed prediction model demonstrated excellent predictive performance (AUC = 0.870).

Compared to previous studies on AL risk factors, such as the prediction model constructed by Huang et al. (AUC = 0.757) and by He et al. (AUC = 0.826), our model demonstrated showed better predictive performance (39, 40). Moreover, this study innovatively proposed the potential mechanisms for the high incidence of cervical AL, which could guide surgeons to evaluate anastomotic blood supply using indocyanine green fluorescence imaging during surgery, enabling intraoperative intervention to prevent AL. Additionally, unlike previous studies that mainly focused on preoperative static indicators, our study incorporated dynamic postoperative monitoring, allowing for more timely and effective interventions. For example, high-risk patients identified by the model may receive oxygen therapy, antibiotics, or maintain enteral tube feeding and delay oral feeding to prevent AL. This comprehensive perioperative monitoring significantly improved the predictive performance (AUC = 0.870) and clinical utility.

We believe that our prediction model has better comprehensiveness and clinical applicability than previous models, helping clinicians implement timely interventions during the perioperative period to reduce AL incidence or alleviate its severity.

Several limitations of this study should be considered when interpreting our findings. First, the single-center design and modest sample size may limit the generalizability of our results and introduce selection bias, although we used consecutive enrolments to minimize this risk. Second, the retrospective nature of the study precludes causal inferences, despite rigorous adjustment for known confounders. Third, although we standardized laboratory timings, biological variability in inflammatory marker kinetics could affect the predictions. Future studies should consider serial measurements on postoperative day 1–3–5–7 to capture dynamic trends. Finally, the modest sample size still presents a potential risk of overfitting. Next, we plan to collaborate with other centers to further validate this risk model using a larger sample size.

## 5 Conclusion

This study confirmed that age, postoperative NLR, postoperative RBC count, and anastomotic location are independent risk factors for AL. We have constructed a risk prediction model for AL after esophagectomy in patients with ESCC, which demonstrates good predictive efficacy, aiding clinicians in the early detection of AL postoperatively and allowing for timely intervention.

Furthermore, we present a detailed summary of anastomotic location, tension, blood perfusion and possible mechanisms of AL, and believe that this will provide new insights for clinicians to avoid the occurrence of AL.

## Data Availability

The raw data supporting the conclusions of this article will be made available by the authors, without undue reservation.
